# Data-driven prognostic factors analysis and personalized follow-up strategies for post-progression survival in locally advanced esophageal squamous cell carcinoma after definitive chemoradiotherapy

**DOI:** 10.1080/07853890.2025.2607188

**Published:** 2026-01-02

**Authors:** Jianjian Qiu, Zhiping Wang, Yuling Ye, Yilin Yu, Mingqiu Chen, Baihua Yang

**Affiliations:** a Department of Radiation Oncology, Clinical Oncology School of Fujian Medical University, Fujian Cancer Hospital, Fuzhou, China; b Department of Radiotherapy, Cancer Center, The First Affiliated Hospital of Fujian Medical University, Fuzhou, Fujian Province, China; c Department of Gynecology, Clinical Oncology School of Fujian Medical University, Fujian Cancer Hospital, Fuzhou, China

**Keywords:** Conditional survival, locally advanced ESCC, personalized follow-up strategies, PPS, prognostic factors

## Abstract

**Background:**

This study investigates clinical characteristics influencing post-progression survival (PPS) in locally advanced esophageal squamous cell carcinoma (ESCC) after definitive chemoradiotherapy (dCRT), aiming to develop individualized follow-up strategies using conditional PPS.

**Methods:**

The correlation between PPS and overall survival (OS) using Spearman correlation analysis. LASSO regression, Cox regression, and machine-learning methods were employed to identify prognostic factors, and a prediction model was constructed. The Shapley additive explanations (SHAP) method was used to interpret the model. Conditional PPS survival rates and recurrence risks were analyzed.

**Results:**

This study enrolled 741 patients, with a median follow-up of 27.2 months. PPS was positively correlated with OS. Prognostic factors included: N stage, tumor length, chemotherapy cycles, platelet-to-albumin ratio, lymphocyte-to-monocyte ratio, age, body mass index, radiotherapy dose, and neutrophil to monocyte to lymphocyte ratio. Calibration curves, decision curves, and ROC curves demonstrated the model’s stability and predictive performance. Subgroup analyses suggested shorter PPS in high-risk patients. After adjusting for other confounders, multi-model analyses continued to show a positive association between the risk score and unfavorable PPS. Conditional PPS analyses across different risk groups revealed that, with increasing survival time, conditional PPS extended correspondingly, and the relapse risk gradually decreased. Finally, individualized follow-up strategies were proposed, indicating intensified monitoring for high-risk patients.

**Conclusion:**

This study fills the research gap in the influencing factors of PPS and personalized follow-up strategies for patients with locally advanced ESCC after dCRT, and provides important clinical evidence for promoting the transformation of post-recurrence management from ‘experience-driven’ to ‘data-driven’.

## Introduction

Esophageal carcinoma (EC) represents one of the most burdensome malignancies worldwide, ranking seventh in terms of global incidence and sixth in mortality among all malignant tumors [1]. For patients with unresectable locally advanced esophageal squamous cell carcinoma (ESCC), definitive chemoradiotherapy (dCRT) is the current standard of care [2]. Despite ongoing refinements in treatment, the prognosis for locally advanced ESCC remains poor, with a global 5-year overall survival (OS) of approximately 15–25% [3]. Notably, about 50–70% of patients experience disease progression within the follow-up period after dCRT, presenting as locoregional relapse and/or distant metastasis [4,5]. This pattern suggests that while initial therapy can control tumor burden to some extent, the biological behavior of disease progression and the therapeutic window thereafter are critical determinants of long-term outcomes. Historically, most clinical investigations have focused on the benefits of first-line therapy and on establishing standardized follow-up strategies. However, systematic evidence regarding treatment options after progression, surveillance strategies, and their outcomes remains relatively scarce. In recent years, post-progression survival (PPS) has emerged as a central metric for evaluating prognosis in this patient population and for guiding subsequent interventions and follow-up planning [6,7].

Imai et al. conducted a study showing that the PPS of patients with locally advanced non-small cell lung cancer who relapsed after receiving first-line radiotherapy and chemotherapy was significantly positively correlated with the OS. Moreover, the Glasgow Prognostic Score and liver metastasis at the time of recurrence were independent prognostic factors affecting PPS [8]. Kobayashi et al. discovered that different imaging progression patterns (such as progression of liver lesions within the liver, progression of liver lesions outside the liver, etc.) in patients with hepatocellular carcinoma who experienced disease progression after receiving immune checkpoint inhibitor treatment were related to PPS [9]. Other studies have also found that advanced age, tumor stage, and lymph node metastasis are associated with shortened PPS [10,11]. PPS not only reflects the biological invasiveness of the tumor to a certain extent, but is also closely related to subsequent salvage treatments (such as surgery, immunotherapy, etc.) and the OS of patients. Through the assessment of PPS, doctors can more accurately grasp the survival trend after progression, optimize individualized treatment decisions, and reasonably arrange the timing of subsequent follow-up and intervention.

Although the clinical significance of PPS has gradually gained attention, studies on PPS after dCRT for locally advanced ESCC, its influencing factors, and its role in individualized follow-up strategies are still insufficient. This research gap limits evidence-driven treatment decisions and resource allocation in multiple aspects: Firstly, there is a lack of systematic comparison of treatment patterns after progression and their impact on survival outcomes; Secondly, there is no unified follow-up rhythm and assessment indicators based on PPS. Therefore, it is necessary to conduct a research framework centered on PPS to reveal the key factors affecting PPS, evaluate the effectiveness of different salvage treatment strategies, and explore individualized follow-up plans, in order to prolong survival while improving quality of life. The purpose of this study is to retrospectively analyze the conditional survival rate of PPS in locally advanced ESCC after dCRT, explore the potential influencing factors of PPS, and propose an individualized follow-up strategy based on PPS to provide scientific basis for follow-up management in clinical practice.

## Materials and methods

### Patients

This study enrolled 741 patients with locally advanced ESCC who underwent dCRT or definitive radiotherapy (dRT) between January 2013 and December 2019. The inclusion criteria were as follows: (1) Pathologically confirmed ESCC; (2) No prior surgical intervention; (3) Tumor stage II–IVA; (4) Absence of other malignant tumor history. The exclusion criteria included: (1) Severe pre-treatment infections or major comorbidities (e.g. hepatic failure, renal failure, severe cardiovascular or cerebrovascular diseases); (2) Incomplete clinical data; (3) Lost to follow-up. The tumor stage of each patient was re-evaluated and finally determined by at least two experienced clinical physicians based on the 8th edition UICC staging system. After screening, 741 patients met the eligibility criteria. This study adhered to the principles of the Declaration of Helsinki and was approved by the Ethics Committee of Fujian Cancer Hospital (K2023-001-01). Clinicopathological data were extracted from electronic medical records, including: age, sex, height, weight, tumor location, tumor length, tumor thickness, T stage, N stage, TNM stage, chemotherapy cycles, chemotherapy regimen, RT dose, as well as routine blood tests and biochemical indicators.

### Treatment

All patients received individualized radiotherapy, primarily employing intensity-modulated radiation therapy (IMRT) or three-dimensional conformal radiotherapy (3D-CRT), with prescribed doses ranging from 50 to 70 Gy delivered in 25–34 fractions. Target volume delineation followed NCCN guidelines: the gross tumor volume (GTV) encompassed primary tumors and involved lymph nodes, while the clinical target volume (CTV) extended at least 3 cm craniocaudally beyond the GTV with an additional 0.5 cm circumferential margin. The planning target volume (PTV) was created by expanding the CTV by 0.5–1 cm, with a 1 cm expansion applied to involved lymph nodes (maintained even without CTV delineation). Treatment objectives mandated ≥95% PTV coverage by the prescribed dose, with all plans adhering to organ-at-risk (OAR) dose–volume constraints.

Regarding chemotherapy, patients received 0–9 cycles of platinum-based regimens categorized into two groups: Group A comprised platinum combination therapies (e.g. paclitaxel/docetaxel with carboplatin, cisplatin, nedaplatin, or lobaplatin), while Group B utilized 5-FU-based protocols (5-FU + cisplatin). Pre- and post-chemotherapy monitoring included complete blood counts, biochemical tests, and electrocardiograms, with dose adjustments in subsequent cycles for significant toxicities. The overall treatment paradigm emphasized individualized approaches, precise target coverage, and rigorous toxicity/safety monitoring to optimize therapeutic efficacy while maintaining safety.

### Calculation of inflammatory and nutritional indicators

Biological rationale for selecting inflammatory indices: during the tumor progression phase, systemic inflammatory responses impact survival outcomes by promoting angiogenesis and suppressing immune function. Systemic immune-inflammation index (SII) = N × PL/L, comprehensively reflects the balance between pro-tumor (neutrophils, platelets) and anti-tumor (lymphocytes) immunity. An elevated SII suggests a tumor microenvironment more conducive to progression. Systemic inflammatory response index (SIRI) = N × M/L, evaluates the balance between myeloid immune cells (neutrophils, monocytes) and lymphocytes, closely associated with tumor-related inflammation. Lymphocyte-to-monocyte ratio (LMR) = L/M, lymphocytes represent adaptive anti-tumor immunity, while monocytes can differentiate into pro-tumor macrophages. This ratio reflects the balance between these two components. Neutrophil to monocyte to lymphocyte ratio (NMLR) = (M + L)/N, provides a comprehensive assessment of the relative proportions of multiple immune cell populations. Neutrophil-to-lymphocyte ratio (NLR) = N/L, a classic inflammatory marker reflecting the state of innate immune activation and adaptive immune suppression. Derived neutrophil to lymphocyte ratio (dNLR) = N/(WBC-L), serves as a surrogate marker providing similar prognostic information when lymphocyte counts are unreliable. Platelet to lymphocyte ratio (PLR) = PL/L, platelets are involved in the metastatic process. This ratio reflects the balance between pro-metastatic potential and immune status.

Biological rationale for selecting nutritional indices: the advanced tumor stage is often accompanied by cachexia and deteriorating nutritional status, directly impacting treatment tolerance and survival. Prognostic nutritional index (PNI) = ALB + 5 × L, Integrally assesses nutritional status (albumin) and immune competence (lymphocytes) A low PNI indicates poor nutritional and immune status. Platelet-to-albumin ratio (PAR) = PL/ALB, combines information on coagulation function (platelets) and nutritional status (albumin). Body mass index (BMI) = W/H^2^, a fundamental indicator of nutritional status. Advanced lung cancer inflammation index (ALI) = BMI × ALB/NLR, uniquely integrates nutritional status (BMI, albumin) and inflammation level (NLR), reflecting the ‘nutrition–inflammation’ balance. Geriatric nutritional risk index (GNRI) = 1.489 × ALB + 41.7 × (W/IW), accurately reflect the discrepancy between actual nutritional status and the ideal state. IW (Ideal weight) = 22 × H^2^, If the actual weight is greater than the ideal weight, then the actual weight/ideal weight = 1. N, neutrophil; PL, platelet; L, lymphocyte; M, monocyte; WBC, white blood cell; ALB, albumin; W, weight; H, height.

### Study endpoints

Study endpoints are divided into primary and secondary endpoints. The primary endpoint is PPS. Secondary endpoints include OS and progression-free survival (PFS). Specific definitions are as follows: PPS refers to the time from tumor progression to death from any cause or to the last follow-up; OS is the time from the start of treatment to death from any cause or to the end of the last follow-up; PFS is the time from the start of treatment to disease progression or to death from any cause or to the last follow-up, whichever occurs first. In our study, disease progression was evaluated based on the Response Evaluation Criteria in Solid Tumors (RECIST) version 1.1, with imaging examinations serving as the primary method for assessment. Clinical symptoms and tumor marker levels were used as supplementary references.

The concept of conditional survival (CS) describes the probability that a patient will remain alive for a future period after receiving standard therapy. For example, if a patient is in a 3-year conditional survival state at 1-year post-treatment, it indicates a likelihood of surviving during the next three years, i.e. the probability of being alive from diagnosis up to year 4. Building on the CS concept, one can derive conditional PPS probabilities to provide a more intuitive view of a patient’s long-term prognosis at specific time points.

### Follow-up strategy

The follow-up plan for all patients is as follows: every 3 months for 1–2 years after treatment, every 6 months for 3–4 years, and every 12 months thereafter. The follow-up assessments include physical examination, laboratory tests (complete blood count, biochemical panel, tumor marker), endoscopic examination (providing direct histopathological information when needed), and imaging studies (cervical, thoracic, and abdominal CT; brain MRI; and PET-CT). Based on clinical judgement, the follow-up frequency should be individualized.

### Statistical analysis

Statistical analyses will be conducted using IBM SPSS Statistics (Version 26) and R software (Version 4.5.2). The optimal cutoff values for the following variables will be determined using receiver operating characteristic (ROC) curve analysis: tumor length, tumor thickness, RT dose, chemotherapy cycles, SII, SIRI, LMR, NMLR, NLR, dNLR, PLR, PNI, PAR, BMI, ALI, and GNRI. Spearman rank correlation analysis will be used to assess the associations among PPS, local-regional recurrence-free survival (LRRFS), distant metastasis-free survival (DMFS), PFS, and OS. Variable selection will be performed using LASSO regression and univariate/multivariate Cox regression analyses, and a predictive model will be constructed based on the selected variables in combination with machine learning methods. We employed a variety of machine learning methods, including decision trees, extreme gradient boosting (XGBoost), generalized linear model (GLM), random forests, and gradient boosting machine (GBM). The training of machine learning models, hyperparameter tuning (using cross-validation and grid search), and validation were conducted on the partitioned training and test sets (with a ratio of 7:3). Performance was evaluated using standard metrics such as AUC and accuracy, and the SHAP method was used for model interpretation. Kaplan-Meier curves will be used to compare survival differences for PPS, PFS, and OS. All analyses will be two-sided, with a significance level set at *p* < 0.05.

## Results

### Clinical characteristics

We ultimately enrolled 741 patients with locally advanced ESCC. Baseline clinical characteristics are shown in Table 1. Of the 741 patients, 473 (63.8%) were aged ≥62 years, 541 (77.9%) were male, 450 (60.7%) received concurrent chemoradiotherapy, 168 (22.7%) received four or more chemotherapy cycles, and 411 (60.7%) had tumors located primarily in the middle to lower thoracic esophagus (61.1%). Tumor stage distribution was II in 150 patients (20.2%), III in 216 (29.1%), and IVA in 375 (50.6%). The optimal cutoff values for the variables (tumor length, tumor thickness, RT dose, chemotherapy cycles, SII, SIRI, LMR, NMLR, NLR, dNLR, PLR, PNI, PAR, BMI, ALI, GNRI) are: 3.60 cm, 1.30 cm, 61 Gy, 4 cycles, 448.82, 0.67, 4.93, 2.10, 1.90, 0.90, 119.57, 51.80, 6.98, 25.54, 375.77, and 103.01.

**Table 1.** Baseline characteristics of 741 cases of locally advanced esophageal squamous cell carcinoma.

Characteristic	No. of patients
Age (years)	
<62	268 (36.2%)
≥62	473 (63.8%)
Sex	
Male	541 (73.0%)
Female	200 (27.0%)
TNM stage	
II	150 (20.2%)
III	216 (29.1%)
IVA	375 (50.6%)
T stage	
T2	48 (6.5%)
T3	355 (47.9%)
T4	338 (45.6%)
N stage	
N0	211 (28.5%)
N1	332 (44.8%)
N2	141 (19.0%)
N3	57 (7.7%)
Tumor location	
Cervical	84 (11.3%)
Upper thoracic	204 (27.5%)
Middle thoracic	74 (10.0%)
Lower thoracic	379 (51.1%)
Tumor length (cm)	
< 3.60	128 (17.3%)
≥ 3.60	613 (82.7%)
Tumor thickness (cm)	
< 1.30	297 (40.1%)
≥ 1.30	444 (59.9%)
Concurrent chemotherapy	
Yes	450 (60.7%)
No	291 (39.3%)
Chemotherapy cycle	
<4	573 (77.3%)
≥4	168 (22.7%)
RT dose (Gy)	
<61	396 (53.4%)
≥61	345 (46.6%)
SII	
<448.82	293 (39.5%)
≥448.82	448 (60.5%)
SIRI	
<0.67	255 (34.4%)
≥0.67	486 (65.6%)
LMR	
<4.93	487 (65.7%)
≥4.93	254 (34.3%)
NLMR	
<2.10	283 (38.2%)
≥2.10	458 (61.8%)
NLR	
<1.90	288 (38.9%)
≥1.90	453 (61.1%)
dNLR	
<0.90	559 (75.4%)
≥0.90	182 (24.6%)
PLR	
<119.57	296 (39.9%)
≥119.57	445 (60.1%)
PNI	
<51.80	562 (75.8%)
≥51.80	179 (24.2%)
PAR	
<6.98	496 (66.9%)
≥6.98	245 (33.1%)
BMI	
<25.54	667 (90.0%)
≥25.54	74 (10.0%)
ALI	
<375.77	370 (49.9%)
≥375.77	371 (49.9%)
GNRI	
<103.01	612 (82.6%)
≥103.01	129 (17.4%)

RT, radiotherapy; SII, systemic immune-inflammation index; SIRI, systemic inflammatory response index; LMR, lymphocyte-to-monocyte ratio; NMLR, neutrophil to monocyte to lymphocyte ratio; NLR, neutrophil to lymphocyte ratio; dNLR, derived neutrophil to lymphocyte ratio; PLR, platelet to lymphocyte ratio; PNI, prognostic nutritional index; PAR, platelet-to-albumin ratio; BMI, body mass index; ALI, advanced lung cancer inflammation index; GNRI, geriatric nutritional risk index.

The median follow-up time was 27.2 months. Progression occurred in 41.4% of patients, and the median PPS was 17.1 months. The median PPS was 31.5 months in the progression-free group and 7.4 months in the progression group, with a statistically significant difference between groups (*p* < 0.05).

### Spearman rank correlation analysis

**Figure 1.**
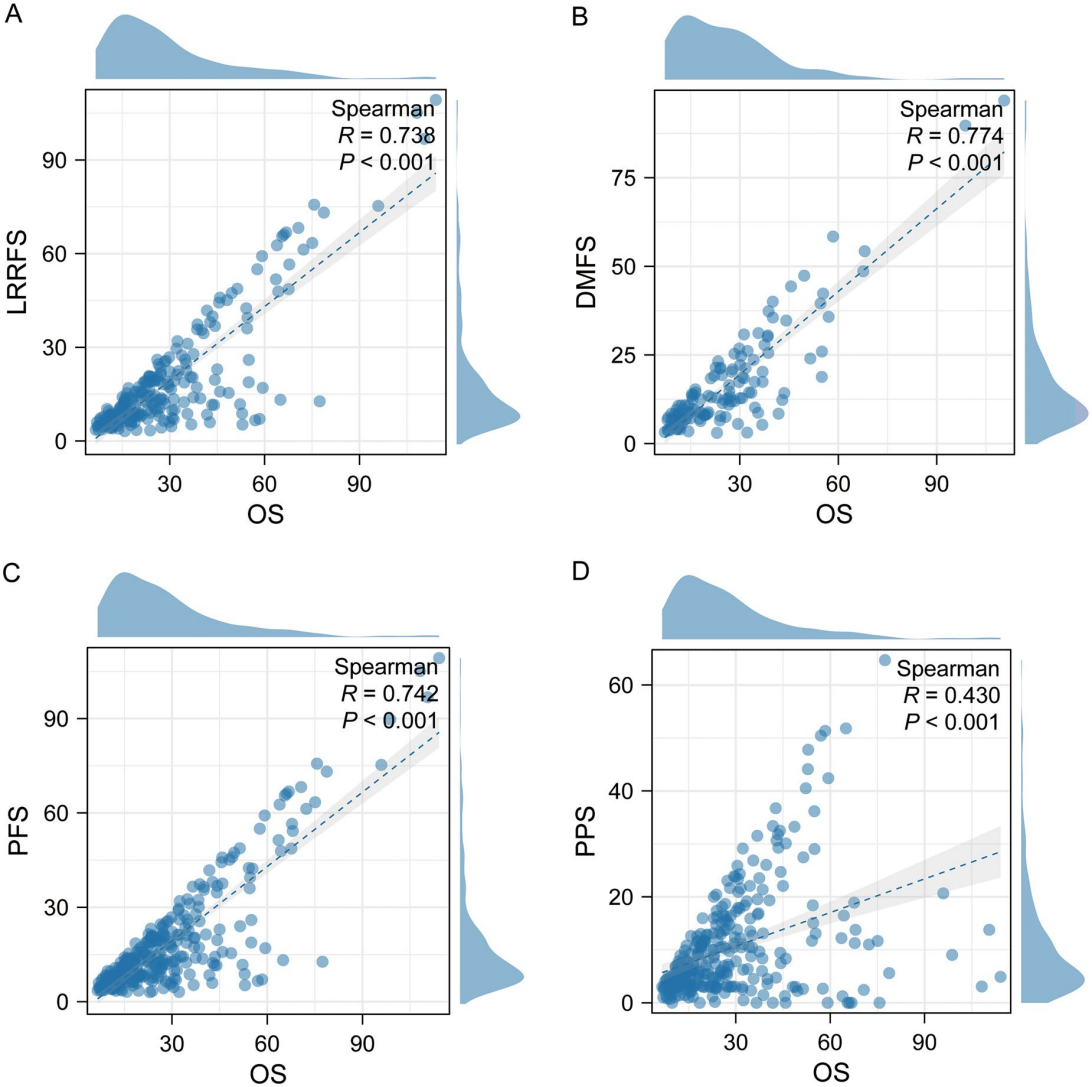
Assess the associations between LRRFS, DMFS, PFS, and PPS and OS using Spearman rank correlation. (A) LRRFS; (B) DMFS; (C) PFS; (D) PPS.

The relationship between each prognostic indicator and OS was evaluated through Spearman rank correlation analysis. The results showed that OS was significantly positively correlated with LRRFS, DMFS, and PFS, with correlation coefficients of respectively *R* = 0.738, 0.774, and 0.742 (*p* < 0.001, Figures 1A–C), while the correlation between OS and PPS was weaker but still significant (*R* = 0.430, *p* < 0.001, Figure 1D).

In summary, PPS is one of the key components of OS, and the two are positively correlated. In cancer treatment, prolonging PPS is one of the important strategies for improving OS.

### Using LASSO regression and Cox regression analysis to screen the clinical characteristics related to PPS

**Figure 2.**
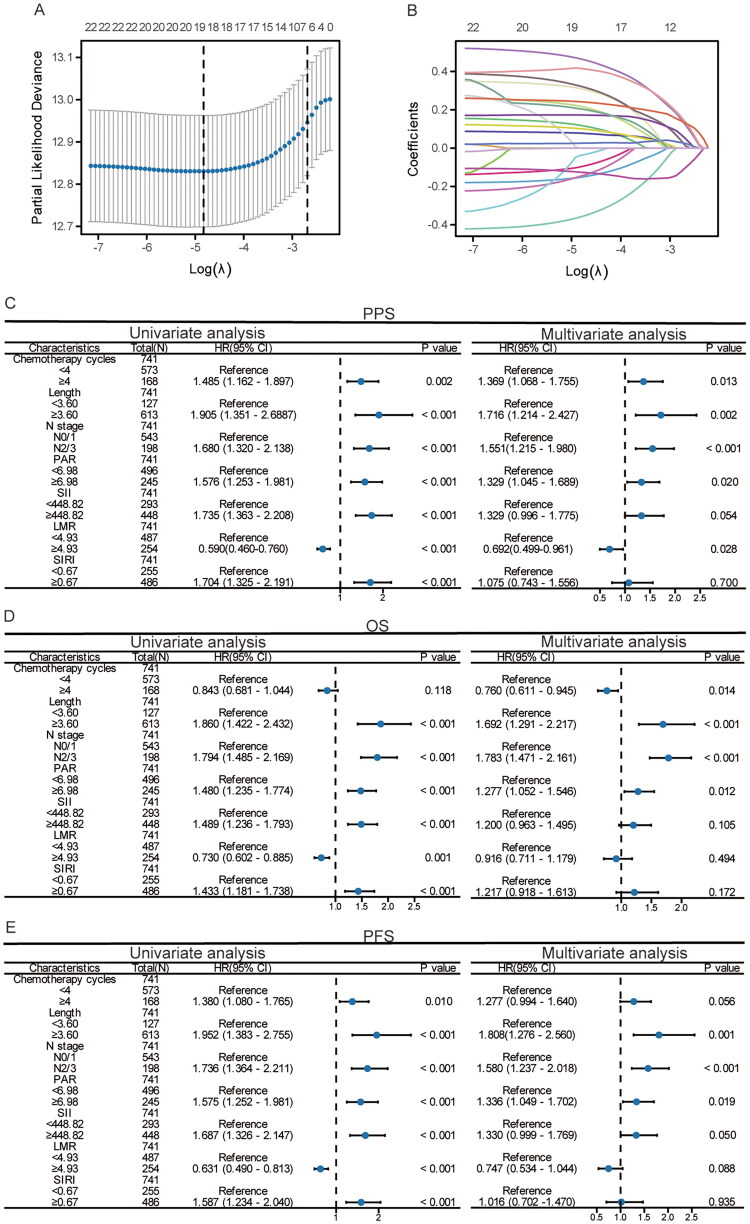
Use LASSO regression to identify the important predictors. (A) The LASSO coefficient path plot reveals seven selected clinical variables. (B) The LASSO coefficient trajectories illustrate the movement of each variable’s coefficient across the regularization path. Cox regression analysis was conducted using these seven variables. (C) PPS; (D) OS; (E) PFS.

This study is based on a retrospective cohort to explore potential clinical factors related to PPS by screening 23 clinical characteristics. Using LASSO regression, seven potential factors were identified from the 23 characteristics: N stage, tumor length, chemotherapy cycles, PAR, LMR, SII, and SIRI (Figures 2A,B). To further delineate their independent associations with PPS, univariate and multivariate Cox regression analyses were performed on the screened features. The multivariate Cox results demonstrated that N stage [(Hazard Ratio), HR], 1.551; 95% Confidence interval (CI), 1.214–2.427; *p* < 0.001], tumor length (HR, 1.716; 95%CI, 1.214–2.427; *p* = 0.002), chemotherapy cycles (HR, 1.369; 95%CI, 1.068–1.755; *p* = 0.013), PAR (HR, 1.329; 95%CI, 1.045–1.689; *p* = 0.020), and LMR (HR, 0.692; 95%CI, 0.499–0.961; *p* = 0.028) constituted independent prognostic factors for PPS (Figure 2C). Furthermore, N stage (HR, 1.783; 95%CI, 1.471–2.161; *p* < 0.001), tumor length (HR, 1.692; 95%CI, 1.291–2.217; *p* < 0.001), chemotherapy cycles (HR, 0.760; 95%CI, 0.611–0.945; *p* = 0.014), and PAR (HR, 1.277; 95%CI, 1.052–1.546; *p* = 0.012) were also independent prognostic factors for OS (Figure 2D); and N stage (HR, 1.580; 95%CI, 1.237–2.018; *p* < 0.001)), tumor length (HR, 1.808; 95%CI, 1.276–2.560; *p* = 0.001), PAR (HR, 1.336; 95%CI, 1.049–1.702; *p* = 0.019), and SII (HR, 1.330; 95%CI, 0.999–1.769; *p* = 0.050) were independent prognostic factors for PFS (Figure 2E). Kaplan–Meier curves visually depicted the association of N stage, tumor length, chemotherapy cycles, PAR, and LMR with patient prognosis (Figure S1). The results demonstrated that higher N stage (Figure S1A), greater tumor length (Figure S1B), fewer than 4 chemotherapy cycles (Figure S1C), PAR ≥6.98 (Figure S1D), and LMR <4.93 (Figure S1E) were significantly correlated with poorer PPS, OS, and PFS.

### Evaluating the importance of clinical features in PPS through machine learning methods

**Figure 3.**
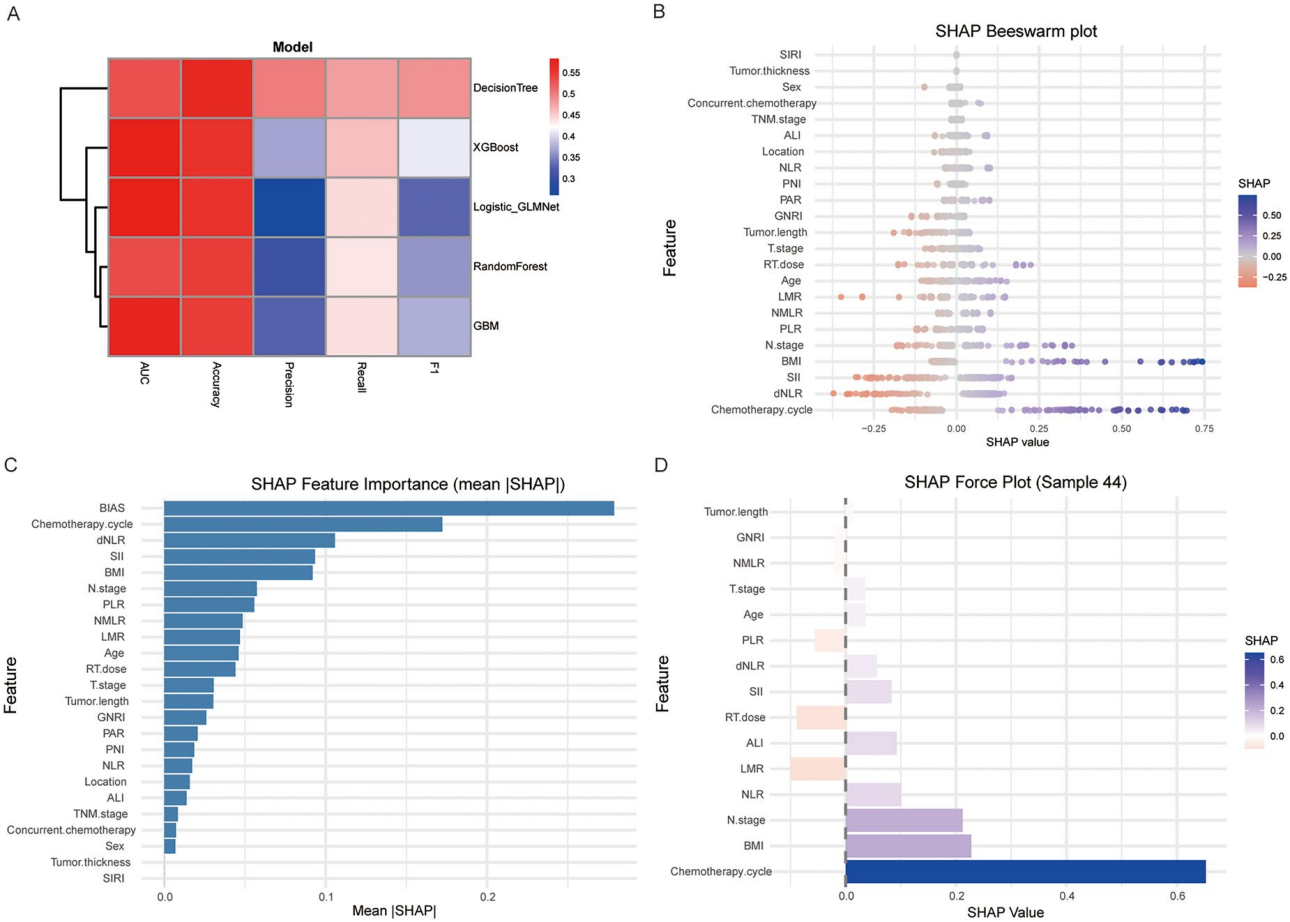
Multivariate feature selection and SHAP analyses for prognosis-related features. (A) Heatmap showing model performance across different algorithms (AUC, accuracy, etc.) (B) SHAP Beeswarm plot illustrating the distribution and impact of top features on model predictions across samples. (C) SHAP Feature Importance ranking the features by their mean absolute SHAP value. (D) SHAP Force Plot for Sample 44, illustrating the per-feature contribution (positive/negative) to the model’s prediction.

This study employed machine learning approaches to further evaluate the importance of clinical features in the predictive model and to identify key variables. We compared five algorithms: decision tree [12], XGBoost [13], GLM [14], random forest [15], and GBM [16]. Model performance was assessed using metrics including AUC, accuracy, precision, recall, and F1 score to determine the most effective predictive model. Results showed that the XGBoost model exhibited the best overall phenotypic predictive capability and was selected for subsequent analyses (Figure 3A).

To interpret the clinical features with the most substantial impact on PPS, we computed SHAP values. SHAP values provide an intuitive visualization of each feature’s contribution to the model output and the direction of its effect, and they allow for visualization-based explanations at the single-sample level. The results indicated that the three strongest predictors associated with an increased risk of adverse PPS outcomes were chemotherapy cycles, N stage, and BMI. Additionally, younger age, higher RT dose, and higher NMLR were also associated with greater PPS risk (Figures 3B,C).

We extracted and visualized individual PPS risk profiles, using waterfall plots to illustrate the positive and negative influence of biomarker contributions on the predictions, thereby constructing individualized PPS risk summaries (Figure 3D).

### Construction of a prognostic nomogram to predict PPS

**Figure 4.**
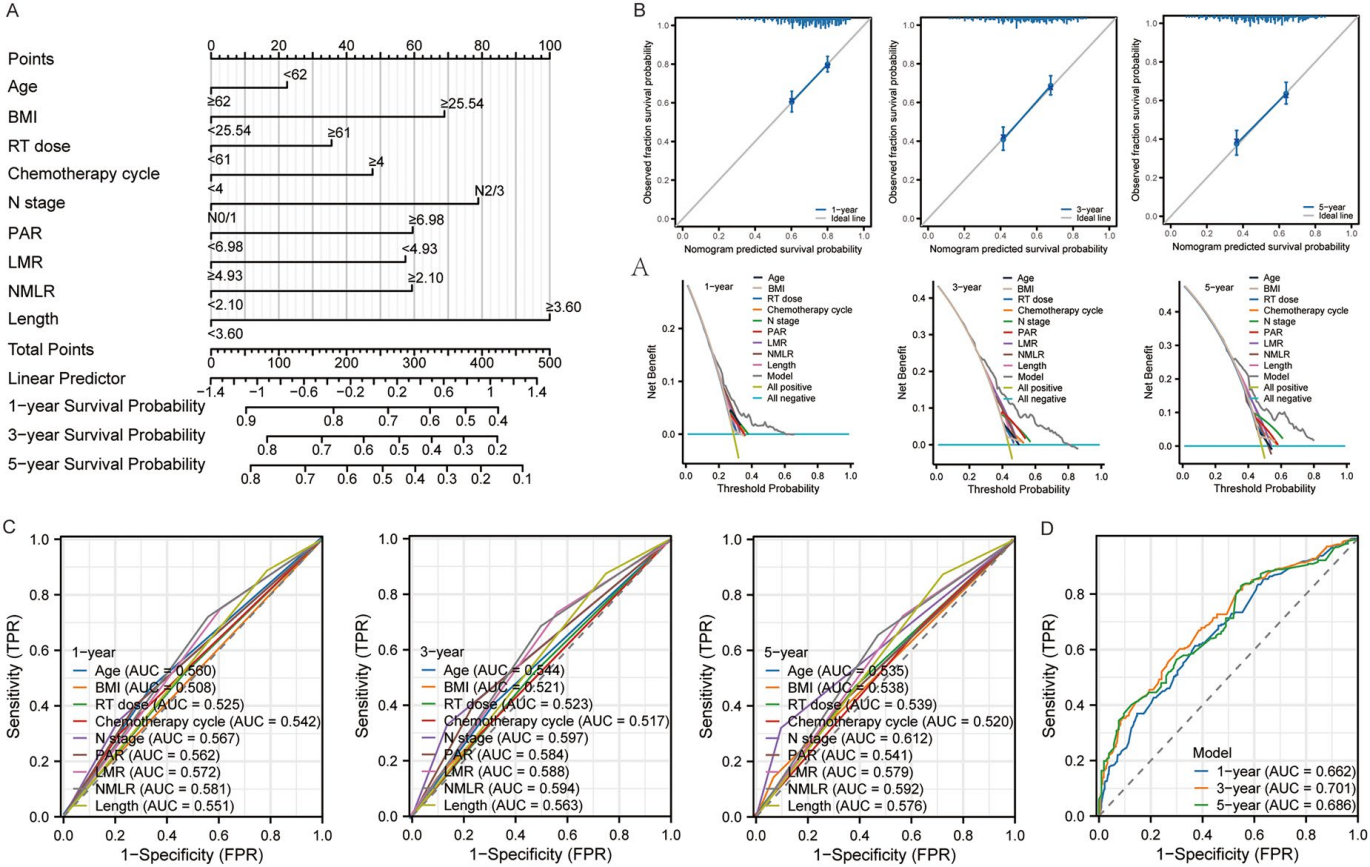
Using LASSO regression, Cox regression and machine learning to determine the most key clinical factors influencing PPS, a nomogram prediction model was constructed and validated. (A) The nomogram model constructed with 9 key clinical factors; (B) Calibration curve to verify the accuracy of the model; (C) Decision curve to evaluate the clinical application value of the model; (D) ROC curve to evaluate the predictive efficacy of each independent variable; (E) ROC curve to evaluate the predictive efficacy of the model.

Based on LASSO regression, Cox regression, and machine learning approaches, we identified nine clinical features significantly associated with PPS in locally advanced ESCC: N stage, tumor length, chemotherapy cycles, PAR, LMR, age, BMI, RT dose, and NMLR. Building on these factors, we developed a PPS prognostic model for locally advanced ESCC using the nine determinants to predict patient outcomes (Figure 4A). Calibration curves demonstrated good agreement between predicted probabilities and observed outcomes at 1, 3, and 5 years, indicating reliable calibration and accuracy of the model (Figure 4B). Decision curve analysis showed that, within the same probability threshold range, the net benefit of the model at 1, 3, and 5 years exceeded that of models using only individual clinical features, suggesting greater clinical utility for decision-making (Figure 4C). Discrimination performance was evaluated using ROC curves and the area under the curve (AUC), revealing that the 1-, 3-, and 5-year model AUCs were higher than those of the individual features, indicating superior predictive capability (Figures 4D–E). In summary, by integrating nine key clinical features with advanced analytical methods, the resulting prognostic model demonstrated improved calibration, decision–analytic value, and predictive performance compared with models based on single features, indicating potential clinical applicability for individualized treatment decisions and risk stratification.

### Risk stratification and subgroup analysis

Based on the nomogram prognostic model, we calculated the total risk score for each patient. The median total risk score in the study cohort was 223, with a range of 0–442. Consequently, patients were categorized into low-risk (0–147), moderate-risk (148–294), and high-risk (295–442) groups. Survival curves demonstrated that the prognosis of the high-risk group was significantly worse than that of the low- and moderate-risk groups (Figure S2). To investigate the impact of different risk groups on common clinicopathological characteristics, subgroup analyses were performed. The results showed that, across comparisons of sex, T stage, TNM stage, tumor location, tumor thickness, and concurrent chemoradiotherapy, the high-risk group had a significantly higher risk of adverse prognosis than the low-risk group (Table 2).

**Table 2.** Subgroup analysis of clinical characteristics in different risk groups.

Variables	*N*	High risk vs moderate risk vs low risk					
		PPS		OS		PFS	
		HR (95% CI)	*P* value	HR (95% CI)	*P* value	HR (95% CI)	*P* value
Sex							
Male	538	1.877 (1.514–2.326)	<0.001	1.557 (1.318–1.814)	<0.001	1.979 (1.597–2.453)	<0.001
Female	203	3.250 (2.154–4.904)	0.009	1.632 (1.203–2.215)	0.002	3.010 (2.005–4.519)	<0.001
T stage							
T2/3	403	2.350 (1.837–3.007)	<0.001	1.633 (1.350–1.975)	<0.001	2.274 (1.785–2.898)	<0.001
T4	338	1.893 (1.412–2.538)	<0.001	1.569 (1.246–1.977)	<0.001	2.120 (1.564–2.873)	<0.001
TNM stage							
II/III	366	2.628 (2.009–3.437)	<0.001	1.571 (1.281–1.927)	<0.001	2.517 (1.933–3.276)	<0.001
Iva	375	1.746 (1.337–2.281)	<0.001	1.644 (1.329–2.034)	<0.001	1.920 (1.458–2.529)	<0.001
Tumor location							
Cervical/upper thoracic	288	2.165 (1.606–2.919)	<0.001	1.651 (1.306–2.087)	<0.001	2.189 (1.627–2.943)	<0.001
Middle/lower thoracic	453	2.192 (1.715–2.801)	<0.001	1.566 (1.297–1.891)	<0.001	2.242 (1.751–2.871)	<0.001
Tumor thickness (cm)							
<1.3	297	2.850 (2.124–3.825)	<0.001	2.039 (1.620–2.565)	<0.001	2.976 (2.222–3.987)	<0.001
≥1.3	444	1.636 (1.264–2.118)	<0.001	1.232 (1.006–1.508)	0.044	1.698 (1.305–2.210)	<0.001
Concurrent chemotherapy							
No	291	2.619 (1.916–3.580)	<0.001	1.751 (1.395–2.198)	<0.001	2.683 (1.978–3.640)	<0.001
Yes	450	1.891 (1.491–2.399)	<0.001	1.513 (1.250–1.831)	<0.001	1.994 (1.566–2.540)	<0.001

PPS, post-progression survival; OS, overall survival; PFS, progression-free survival.

### The relationship between risk scores and adverse prognosis

The relationship between the risk score and adverse outcomes is shown in Table 3. The incidences of adverse events for PPS across the low-, moderate-, and high-risk groups were 22.82%, 41.10%, and 65.83%, respectively. Similarly, the incidences of adverse events for OS and PFS increased with higher risk scores, indicating a significant positive correlation between the risk score and unfavorable prognosis.

**Table 3.** Relationship between risk scores and adverse PPS.

					HR (95% CI), *P* value		
					Crude model	Minimally adjusted model	Fully adjusted model
					(Model 1)	(Model 2)	(Model 3)
PPS	Categories	*N* (total)	*N* (events)	Percentage			
	Low	149	34	22.82%	Reference	Reference	
	Moderate	472	194	41.10%	2.302 (1.598–3.316),<0.001	2.113 (1.454–3.070),<0.001	1.950 (1.326–2.867),0.001
	High	120	79	65.83%	4.806 (3.207–7.202),<0.001	4.193 (2.761–6.366),<0.001	3.659 (12.353-5.691),<0.001
	P trend				<0.001	<0.001	<0.001
OS	Low	149	71	47.65%	Reference	Reference	
	Moderate	472	324	68.64%	1.829 (1.415–2.365),<0.001	1.553 (1.192–2.023),0.001	1.430 (1.088–1.879),0.010
	High	120	99	82.50%	2.640 (1.944–3.586),<0.001	2.214 (1.618–3.029),<0.001	2.017 (1.454–2.799),<0.001
	P trend				<0.001	<0.001	<0.001
PFS	Low	149	34	22.82%	Reference	Reference	
	Moderate	472	194	41.10%	2.359 (1.637–3.398),<0.001	2.100 (1.445–3.051),<0.001	1.879 (1.276–2.767),0.001
	High	120	79	65.83%	5.070 (3.381–7.601),<0.001	4.329 (2.854–6.565),<0.001	3.716 (2.395–5.767),<0.001
	P trend				<0.001	<0.001	<0.001

PPS, post-progression survival; OS, overall survival; PFS, progression-free survival.

Across different models, the estimated effects of adverse prognosis are as follows: in the crude, unadjusted model, the moderate-risk group had a 130.2% higher PPS adverse prognosis risk compared with the low-risk group, and the high-risk group had a 380.6% higher PPS adverse prognosis risk. In Model 2, which adjusted for sex, T stage, TNM stage, tumor location, tumor thickness, and concurrent chemoradiotherapy, the moderate-risk and high-risk groups showed a 111.3% and 319.3% higher PPS adverse prognosis risk, respectively, relative to the low-risk group. In Model 3, further adjusting for indices such as SII, SIRI, NLR, dNLR, PLR, PNI, ALI, and GNRI, the corresponding increases were 95.0% and 265.9%. The same trend was observed in multivariable analyses for OS and PFS, indicating that, regardless of variable adjustments, both the moderate- and high-risk groups had a significantly higher risk of adverse prognosis, further confirming a significant positive association between the risk score and adverse outcomes.

### Developing individualized follow-up strategies based on PPS for conditional survival and recurrence risk

**Figure 5.**
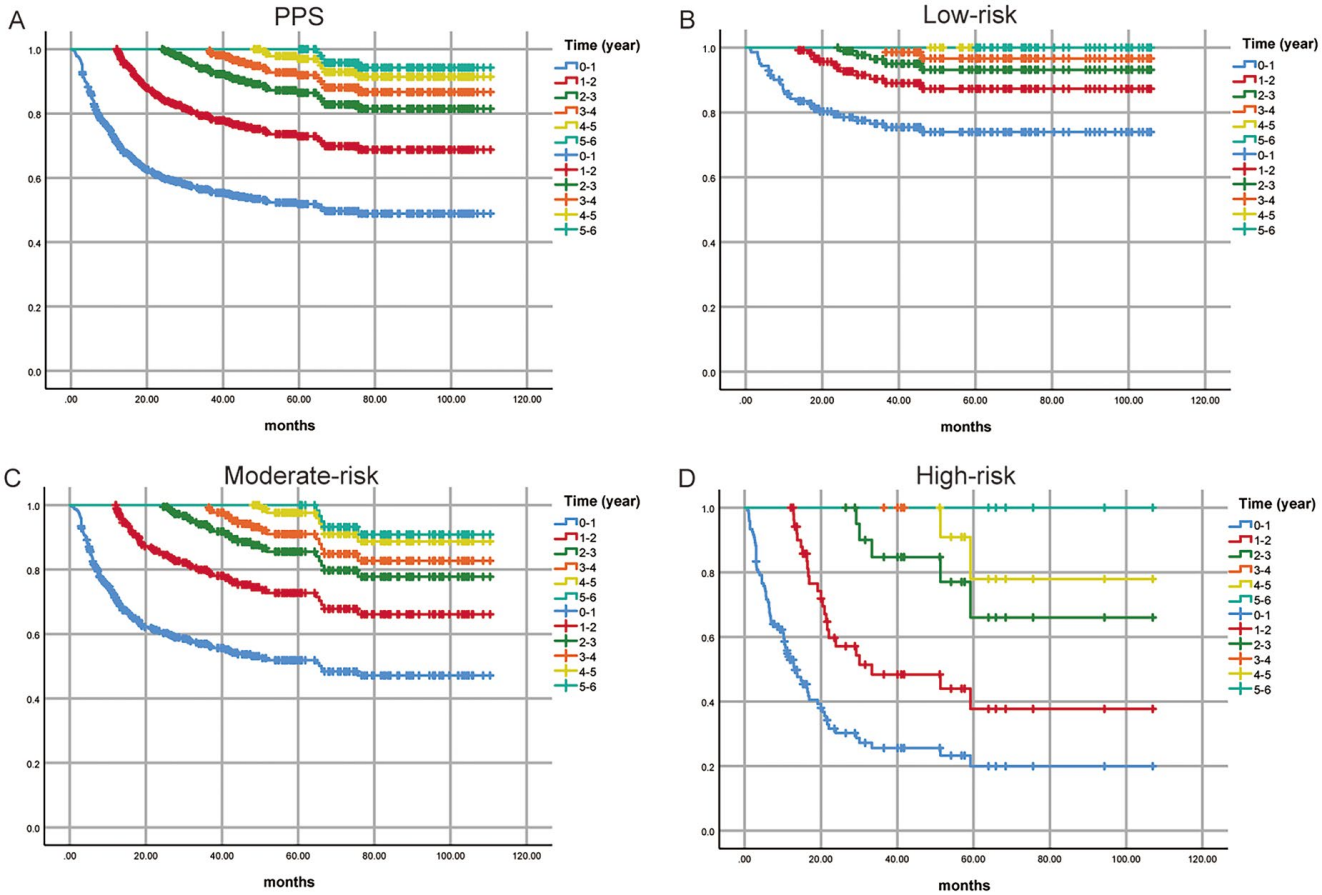
Conditional survival. (A) The overall conditional survival probability of PPS. (B) The conditional survival probability of PPS in the low-risk group. (C) The conditional survival probability of PPS in the moderate-risk group. (D) The conditional survival probability of PPS in the high-risk group.

**Figure 6.**
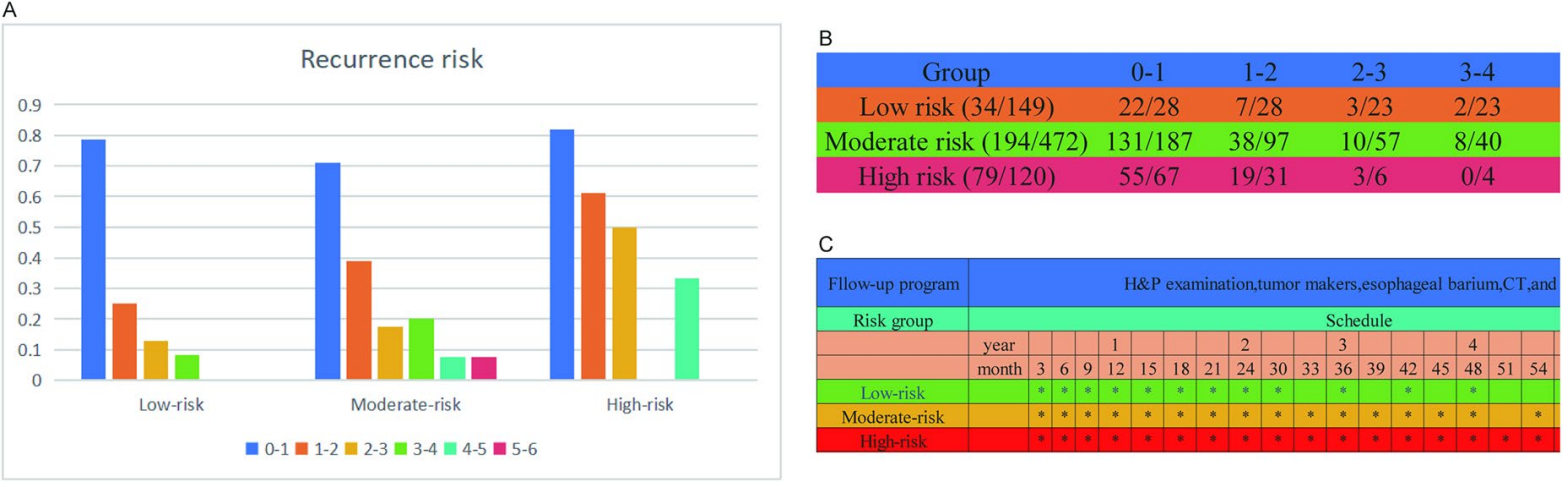
Recurrent risk and individualized follow-up strategies. (A) The bar chart shows the recurrence probabilities of different risk groups in different years. (B) The table shows the number of recurrences in different years for different risk groups. (C) Individualized follow-up strategies based on the recurrence probabilities of different risk groups.

This study has demonstrated that the PPS risk score is closely related to adverse prognosis. After identifying the high-risk factors for PPS, to optimize the management of patients with progression, we performed conditional PPS and recurrence risk analyses and developed individualized follow-up strategies to enhance follow-up effectiveness. As shown in Figure 5A, the five-year survival rates for PPS were 51.9%; the conditional five-year survival rates after 1, 2, and 3 years were 72.9%, 86.4%, and 91.9%, respectively, indicating that longer survival is associated with extended PPS. The same trend was observed in the conditional PPS across different risk groups (Figures 5B–D). Figure S3A shows that the recurrence risk of PPS decreases progressively over time, with similar patterns across risk groups (Figures S3B–D). Further stratified annual recurrence risks by time interval were as follows: Low-risk group: 0–1 year 78.6%; 1–2 years 25.0%; 2–3 years 13.0%; 3–4 years 8.7%; 4–5 years and 5–6 years 0%. Moderate-risk group: 0–1 year 70.1%; 1–2 years 39.2%; 2–3 years 17.5%; 3–4 years 20.0%; 4–5 years 7.7%; 5–6 years 7.7%. High-risk group: 0–1 year 82.1%; 1–2 years 61.3%; 2–3 years 50.0%; 4–5 years 33.3%; 3–4 years and 5–6 years were not estimable due to small sample sizes. Based on risk extrapolation, the recurrence risk in the high-risk group is anticipated to exceed 15% during the 3–4 and 5–6 years intervals (Figures 6A,B). On the basis of these recurrence risk assessments, we formulated individualized follow-up strategies: follow-up every 3 months when the annual recurrence risk exceeds 15%; every 6 months for 5–15%; and annually when it is below 5%. The specific follow-up schedules for each risk group are depicted in Figure 6C.

## Discussion

Among patients with locally advanced ESCC who have received dCRT, disease recurrence remains the predominant mode of treatment failure [17,18]. It imposes substantial psychological and physiological burdens on patients and significantly impacts their quality of life and subsequent treatment options. Existing studies predominantly focus on short-term outcomes such as treatment response, local control rate, or OS, lacking systematic evidence for a multi-dimensional comprehensive assessment of factors influencing PPS. PPS is used to measure patients’ survival performance after disease progression and is directly related to the individualization of follow-up strategies and resource allocation. We first performed Spearman correlation analyses and found that, in addition to LRRFS, DMFS, and PFS being closely related to OS, PPS also shows a significant positive correlation with OS. Based on this, we subsequently employed a combination of methods—LASSO regression, Cox regression, and machine learning approaches—to identify prognostic factors associated with PPS, and we developed a nomogram model for predicting PPS prognosis. The main findings of the study are as follows: The N stage, tumor length, chemotherapy cycles, PAR, LMR, age, BMI, RT dose, and NMLR are important prognostic factors for PPS. After incorporating the above variables into the model, the reliability and stability of the model are confirmed through calibration curve, decision curve, and ROC curve validation. Further risk stratification and subgroup analysis show that the PPS of the high-risk group is significantly worse. After adjusting for confounding factors, multi-model analysis showed a positive correlation between risk scores and poor prognosis. In the conditional PPS analysis, as the survival time increases, the patients’ PPS also extends accordingly, and the recurrence risk shows a downward trend. Based on the recurrence risk, an individualized follow-up strategy is proposed: when the annual recurrence risk exceeds 15%, follow-up every 3 months is recommended; when the recurrence risk is between 5% and 15%, follow-up every 6 months is recommended; when the recurrence risk is less than 5%, the follow-up frequency is reduced to once a year. This strategy aims to optimize resource allocation, reduce unnecessary follow-up burdens, and increase the monitoring intensity and intervention opportunities for high-risk patients.

Numerous studies have demonstrated a positive correlation between PPS and OS [7,19,20]. Due to variations in tumor biology, genomic profiles, and the tumor microenvironment, different types of solid tumors often exhibit distinct progression patterns, which are closely associated with survival following disease progression. These progression patterns have been shown to exert varying impacts on patient prognosis. Specifically, Jonas et al. categorized progression patterns into three risk groups: low-risk (progression of existing lesions only), moderate-risk (appearance of new lesions without progression of existing ones), and high-risk (progression of existing lesions accompanied by the emergence of new lesions). This stratification has been found to be significantly correlated with PPS across various solid tumors [21]. Yao et al. classified progression as either early recurrence (occurring within two years of treatment) or late recurrence (more than two years post-treatment), identifying both as independent prognostic factors for survival after recurrence [22]. Furthermore, the initial site of tumor recurrence has also been shown to influence PPS. Laska et al. reported that bone metastasis and abdominal organ recurrence (e.g. in the liver or adrenal glands) are independent adverse prognostic indicators in lung cancer, significantly reducing survival after recurrence [23]. In patients with resectable esophageal cancer, distant recurrence and involvement of more than three sites have been independently linked to poorer recurrence-free survival [24]. Lastly, the therapeutic approach following recurrence has also been identified as an independent factor influencing PPS, highlighting the critical role of treatment selection in determining survival outcomes [25].

The factors influencing the post-recurrence survival period remain to be further elucidated. Using LASSO regression, Cox regression, and machine learning techniques, we identified several variables significantly associated with poorer PPS: age <62 years, N2/3 stage, primary tumor length ≥3.60 cm, chemotherapy cycles ≥4 cycles, RT dose ≥61 Gy, BMI ≥25.54, PAR ≥6.98, LMR <4.93, and NMLR ≥2.10. Patients exhibiting these characteristics tended to have shorter PPS. The N stage serves as a direct indicator of lymph node metastasis. An increase in the N stage indicates a higher risk of tumor recurrence [26]. Tumor length, serving as a surrogate marker of primary tumor burden, was significantly correlated with shorter PPS, indicating that the aggressiveness of the primary tumor remains a critical driver of disease progression after recurrence [27,28]. The association between prolonged chemotherapy cycles and reduced PPS highlights the need for a balanced approach in clinical practice. Rather than increasing the number of chemotherapy cycles indiscriminately, treatment intensity, toxicity, and patient-specific benefits should be carefully evaluated. Furthermore, the significant relationship between inflammatory and nutritional indicators (PAR, LMR, NMLR) and PPS represents a key finding of this study, underscoring the regulatory role of the tumor microenvironment in post-recurrence prognosis. Elevated PAR levels reflect systemic inflammation and malnutrition, suggesting that the imbalance between inflammatory and nutritional status plays a pivotal role in disease progression [29]. A decreased LMR indicates an immunosuppressive state, wherein pro-inflammatory cytokines secreted by monocytes may facilitate tumor cell proliferation and metastasis [30,31]. An increased NMLR, as a composite inflammatory marker, may reflect neutrophil-mediated immune evasion and monocyte-induced remodeling of the tumor microenvironment [32]. Based on these variables, we developed a nomogram model to predict PPS. The model’s reliability and stability were validated using calibration curves, decision curves, and ROC curves. Each patient was assigned a risk score and stratified accordingly, revealing a significant positive correlation between risk score and PPS. Following subgroup analysis and adjustment for potential confounding factors, the model demonstrated robust clinical applicability.

Currently, the follow-up strategies for patients with locally advanced ESCC after dCRT are mostly based on the recommendations of guidelines (such as NCCN, ESMO and ASCO) [33–35]. Typically, imaging examinations and tumor marker monitoring are conducted every 3–6 months. However, there are few clinical studies on the follow-up strategies after recurrence. Moreover, the existing strategies adopt a one-size-fits-all approach, failing to fully consider individual differences and clarify key issues such as ‘which patients need enhanced follow-up’ and ‘how to adjust the follow-up intervals’. This leads to strong subjectivity in follow-up strategies in clinical practice and a lack of evidence-based support. This study, based on the risk stratification of conditional PPS, analyzes the recurrence risks of different risk groups at various time intervals and proposes individualized follow-up intensities and intervals: Low-risk group: The recurrence risk is higher than 15% from 0 to 2 years, and it is recommended to conduct follow-up every 3 months; the recurrence risk is between 5% and 15% from 2 to 4 years, and follow-up every 6 months is recommended; the recurrence risk is less than 5% from 4 to 6 years, and annual follow-up is recommended. Moderate-risk group: The recurrence risk is higher than 15% from 1 to 4 years, and follow-up every 3 months is recommended; the recurrence risk is between 5% and 15% from 4 to 6 years, and follow-up every 6 months is recommended. High-risk group: The recurrence risk is higher than 15% from 1 to 6 years, and follow-up every 3 months is recommended. Under this premise, the individualized follow-up strategy not only addresses the key issues of ‘when to conduct follow-up’ and ‘follow-up frequency’ but also more efficiently allocates clinical resources and enhances the monitoring intensity and effectiveness for high-risk populations.

The primary clinical value of our model extends beyond its statistical performance to its capacity for risk stratification and guidance of individualized management in patients with recurrent ESCC. By calculating a risk score for each individual, clinicians can objectively categorize patients into distinct prognostic subgroups, thereby directly informing therapeutic decision-making following disease recurrence. For high-risk patients identified by the model, who are anticipated to have shorter post-progression survival, it facilitates earlier prognostic disclosure and discussions on palliative care goals. More importantly, the results provide a clear indication for initiating multidisciplinary team consultation to consider more aggressive treatment regimens, such as combination immunotherapy or enrollment in clinical trials, in an effort to alter the disease trajectory. For low-risk patients with a more favorable predicted survival, the model supports the adoption of a ‘watch and wait’ strategy, while concurrently emphasizing the necessity of sustained supportive care and quality of life maintenance. Furthermore, the model can be integrated with the proposed risk-adapted follow-up strategy to form a closed-loop management system: the radiological detection of recurrence serves not merely as an endpoint of surveillance, but as a critical trigger for initiating stratified treatment interventions. For example, the intensive 3-month follow-up recommended for the high-risk group aims to identify asymptomatic recurrence at the earliest possible stage, thereby creating a critical time window for the timely administration of second-line therapy. This synergy between monitoring and intervention underscores the practical utility of our model in optimizing resource allocation and achieving fully personalized management throughout the continuum of care—from follow-up scheduling to therapeutic execution—potentially influencing overall survival outcomes.

This study benefits from a large-scale and well-characterized patient cohort, which enables robust multivariate analysis and the control of potential confounding factors. Despite our efforts, it is necessary to acknowledge that this study has certain limitations. Firstly, retrospective studies inherently carry biases that are difficult to fully eliminate. Secondly, this study collected PPS-related information from electronic medical records, which may be limited by missing key variables such as relapse patterns and post-relapse treatment strategies, thereby affecting the completeness and interpretability of the results. Although variable selection and model stability were enhanced using methods such as LASSO regression, Cox regression, and multimodal machine learning, external validation remains insufficient. Moreover, the model uses conditional PPS for risk stratification and provides recommendations on follow-up frequency; the external applicability of these conclusions across different treatment contexts and diverse patient populations requires further validation. To improve external validity, future work should undertake multicenter prospective studies to perform external validation and to evaluate the applicability and clinical value of the model across various treatment regimens and patient characteristics.

## Conclusion

This study not only provides a reliable model tool for the prognosis assessment of PPS, but also promotes the precise process of individualized management of PPS in ESCC patients after dCRT through the clinical transformation of ‘indicators–strategies’, filling the gap in this field.

## Supplementary Material

Supplemental Material

## Data Availability

The data supporting the results of this study can be provided by the corresponding author upon reasonable request.

## Abbreviation

EC: esophageal carcinoma
ESCC: esophageal squamous cell carcinoma
dCRT: definitive chemoradiotherapy
OS: overall survival
PPS: post-progression survival
dRT: definitive radiotherapy
IMRT: intensity-modulated radiation therapy
3D-CRT: three-dimensional conformal radiotherapy
GTV: gross tumor volume
CTV: clinical target volume
PTV: planning target volume
OAR: organ-at-risk
SII: systemic immune-inflammation index
SIRI: systemic inflammatory response index
LMR: lymphocyte-to-monocyte ratio
NMLR: neutrophil to monocyte to lymphocyte ratio
NLR: neutrophil to lymphocyte ratio
dNLR: derived neutrophil to lymphocyte ratio
PLR: platelet to lymphocyte ratio
PNI: prognostic nutritional index
PAR: platelet-to-albumin ratio
BMI: body mass index
ALI: advanced lung cancer inflammation index
GNRI: geriatric nutritional risk index
PFS: progression-free survival
CS: conditional survival
ROC: receiver operating characteristic
LRRFS: local-regional recurrence-free survival
DMFS: distant metastasis-free survival
SHAP: shapley additive explanations
HR: hazard ratio
CI: confidence interval
XGBoost: extreme gradient boosting
GLM: generalized linear model
GBM: gradient boosting machine
AUC: the area under the curve
