# Supplementary material for: Data-driven prognostic factors analysis and personalized follow-up strategies for post-progression survival in locally advanced esophageal squamous cell carcinoma after definitive chemoradiotherapy
Source: Ann Med. 2026 Jan 2;58(1):2607188. doi: 10.1080/07853890.2025.2607188 (PMC12777853; doi:10.1080/07853890.2025.2607188)
Supplement: Supplemental Material [file IANN_A_2607188_SM7489.zip › Fig_Suppl/Fig caption.docx]

**FigureS1** The survival differences analysis. (A) N stage; (B) Tumor length; (C) Chemotherapy cycles; (D) LMR; (E) PAR.

**FigureS2** The KM curve shows the survival differences of the different risk groups. (A) the low-risk group; (B) the moderate-risk group; (C) the high-risk group.

**FigureS3** The recurrence risks of different risk groups. (A) The overall recurrence risks of PPS. (B) the low-risk group. (C) the moderate-risk group. (D) the high-risk group.
