# Supplementary figures and images for: Data-driven prognostic factors analysis and personalized follow-up strategies for post-progression survival in locally advanced esophageal squamous cell carcinoma after definitive chemoradiotherapy
Source: Ann Med. 2026 Jan 2;58(1):2607188. doi: 10.1080/07853890.2025.2607188 (PMC12777853; doi:10.1080/07853890.2025.2607188)

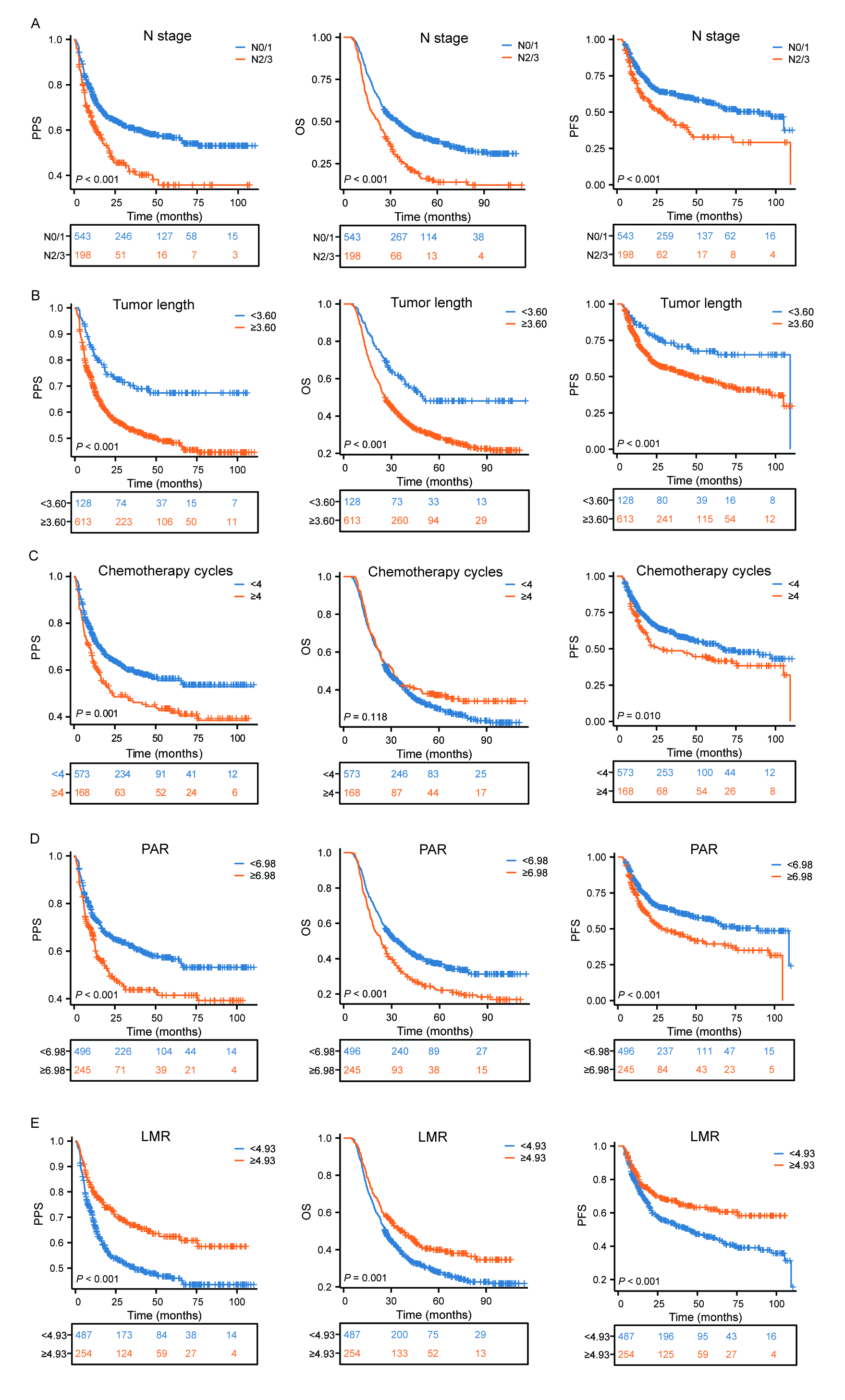

Supplement: Supplemental Material [file IANN_A_2607188_SM7489.zip › Fig_Suppl/Figure S1.tif]

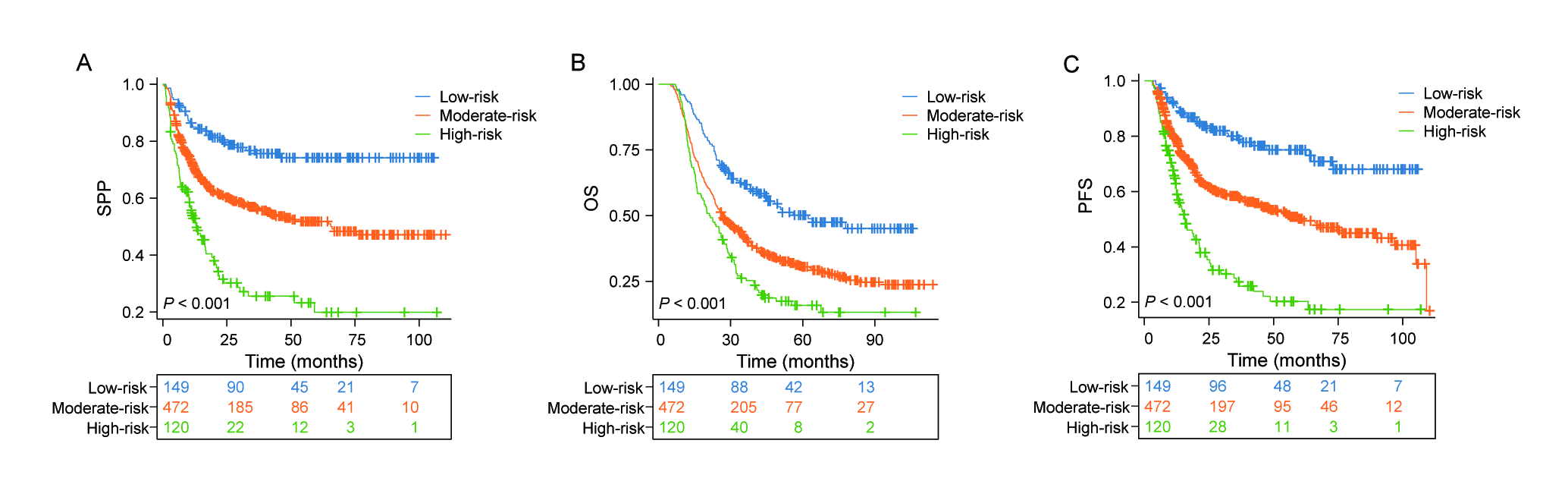

Supplement: Supplemental Material [file IANN_A_2607188_SM7489.zip › Fig_Suppl/Figure S2 (2).tif]

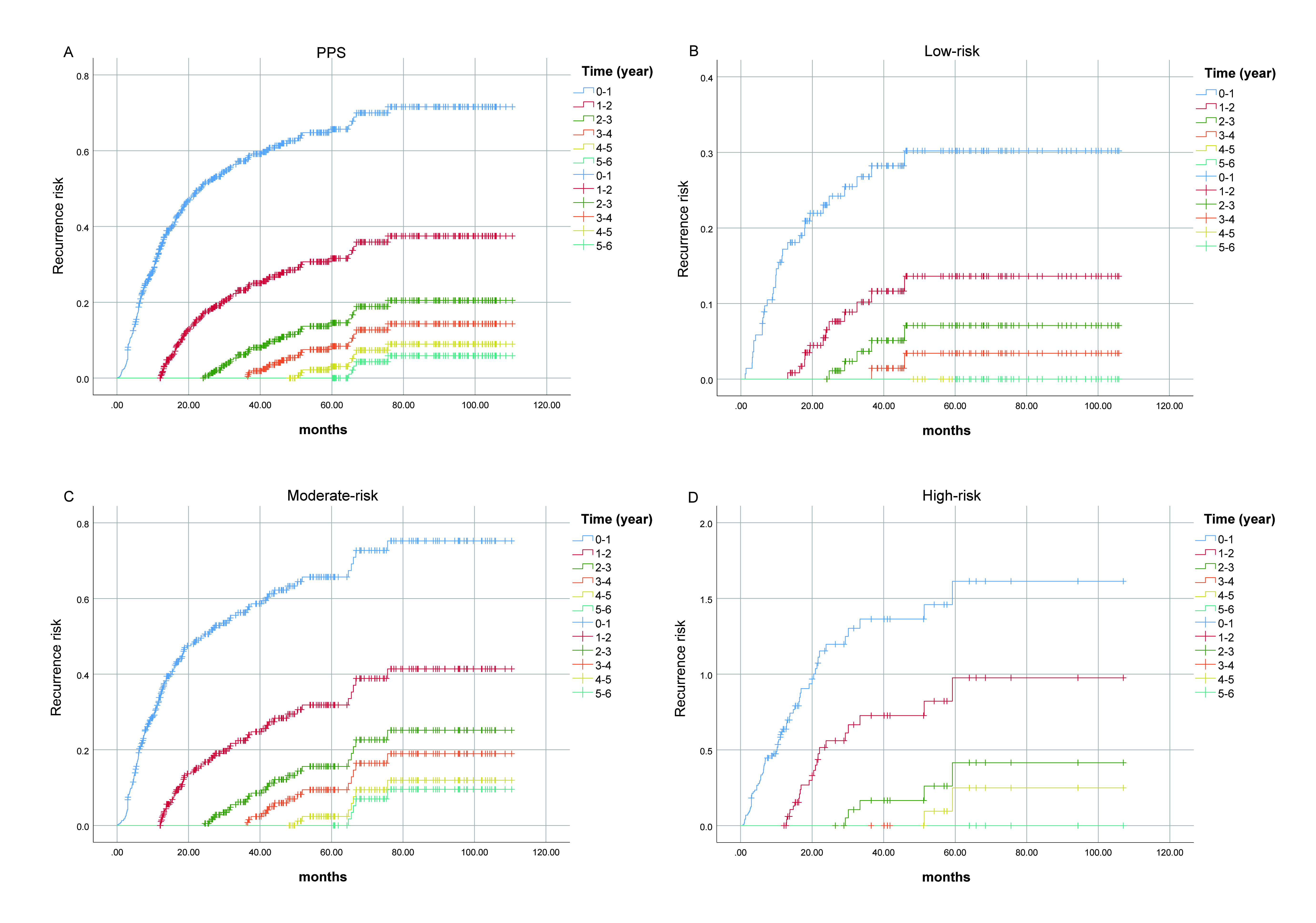

Supplement: Supplemental Material [file IANN_A_2607188_SM7489.zip › Fig_Suppl/Figure S3.tif]
